# Paucigranulocytic Asthma in Aspirin‐Hypersensitive Patients: Pleiotropic Regulation of Type 2 Biomarkers

**DOI:** 10.1002/clt2.70186

**Published:** 2026-07-19

**Authors:** Radosław Kacorzyk, Piotr Szatkowski, Agnieszka Gawlewicz‐Mroczka, Adam Ćmiel, Anna Gielicz, Adam Stępień, Patryk Hartwich, Marek Sanak, Lucyna Mastalerz

**Affiliations:** ^1^ 2nd Department of Internal Medicine Jagiellonian University Medical College Krakow Poland; ^2^ Doctoral School of Medical and Health Sciences Jagiellonian University Medical College Krakow Poland; ^3^ Department of Applied Mathematics AGH University of Science and Technology Krakow Poland; ^4^ Department of Otolaryngology Jagiellonian University Medical College Krakow Poland

**Keywords:** cluster analysis, induced sputum, nonsteroidal anti‐inflammatory drug–exacerbated respiratory disease, paucigranulocytic inflammatory phenotype

## Abstract

**Background:**

Up to 50% of patients with nonsteroidal anti‐inflammatory drug‐exacerbated respiratory disease (N‐ERD) exhibit a noneosinophilic airway inflammatory phenotype, with paucigranulocytic asthma being the most prevalent. The aim was to identify clusters within N‐ERD and aspirin‐tolerant asthma (ATA) controls with a paucigranulocytic asthma phenotype.

**Methods:**

Two separate hierarchical cluster analyses were performed using 23 variables in 36 N‐ERD patients and 19 ATA controls. Variables included demographic, clinical, treatment‐related, and hematologic parameters; sputum cytology; sinus computed tomography findings; and eicosanoid levels in induced sputum supernatant (ISS) and urine.

**Results:**

Two clusters were identified in each group: 1_N‐ERD_, 2_N‐ERD_, 1_ATA_, and 2_ATA_. Cluster 1_N‐ERD_ patients had less severe asthma and required lower doses of inhaled corticosteroids (ICS) but showed higher blood eosinophil counts, Lund‐Mackay (LM) scores, and ISS leukotriene E_4_ (LTE_4_) levels than those in cluster 2_N‐ERD_. Cut‐off values defining a T2‐high paucigranulocytic asthma profile in N‐ERD included LM score ≥ 18, blood eosinophils ≥ 300 cells/mm^3^, and ISS LTE_4_ ≥ 30 pg/mL, with the LM score demonstrating the highest AUC at 0.8. Among ATA controls, cluster 1_ATA_ had more severe asthma, higher ICS doses, more severe sinonasal disease, and increased ISS leukotriene D_4_ and LTE_4_ levels compared with cluster 2_ATA_.

**Conclusion:**

Two clusters were identified among N‐ERD patients with a paucigranulocytic asthma phenotype. Cluster 1_N‐ERD_ was characterized by milder asthma, more severe sinonasal disease, elevated blood eosinophil counts, and elevated ISS LTE_4_. This T2‐high‐like cluster may represent a subgroup requiring further evaluation for anti‐T2 biologic therapy targeting chronic rhinosinusitis with nasal polyps, although further validation is required.

## Graphical abstract

Two clusters were identified among N‐ERD patients with a paucigranulocytic airway inflammatory phenotype. Cluster 1_N‐ERD_ showed milder asthma but higher blood eosinophil counts, more severe sinonasal disease, and elevated LTE_4_ levels in induced sputum supernatant. Severe sinonasal disease and elevated local LTE_4_ may indicate type 2 inflammation despite paucigranulocytic sputum. ATA, aspirin‐tolerant asthma; EOS, eosinophil; IgE, immunoglobulin E; LTE_4_, leukotriene E4; N‐ERD: nonsteroidal anti‐inflammatory drug–exacerbated respiratory disease.

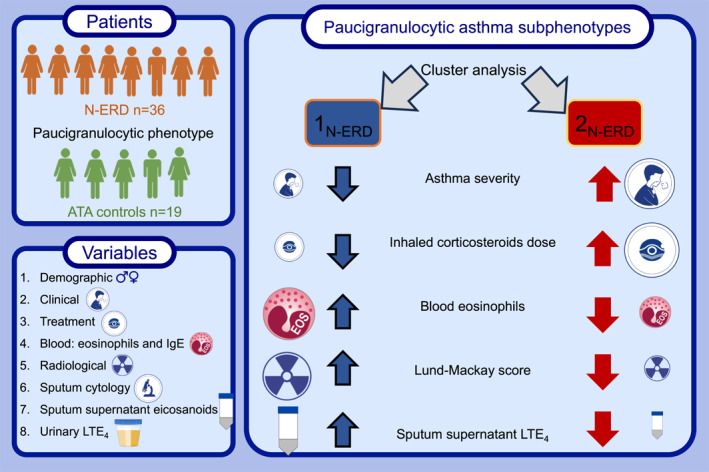

**Abbreviations**
5‐LO: 5‐lipoxygenase
ATA: aspirin‐tolerant asthma
COX‐1: cyclooxygenase type 1
CRSwNP: chronic rhinosinusitis with nasal polyps
CT: computed tomography
FEV_1_
: forced expiratory volume in one second
HPLC‐MS/MS: high‐performance liquid chromatography–tandem mass spectrometry
ICS: inhaled corticosteroids
ISS: induced sputum supernatant
LM score: Lund–Mackay score
LTC_4_
: leukotriene C_4_
LTD_4_
: leukotriene D_4_
LTE_4_
: leukotriene E_4_
N‐ERD: nonsteroidal anti‐inflammatory drug–exacerbated respiratory disease
PGD_2_
: prostaglandin D_2_
PGE_2_
: prostaglandin E_2_
SNOT‐22: 22‐item Sino‐Nasal Outcome Test

## 1 Introduction

Nonsteroidal anti‐inflammatory drug–exacerbated respiratory disease (N‐ERD) is a chronic inflammatory condition characterized by a triad of asthma, chronic rhinosinusitis with nasal polyps (CRSwNP), and hypersensitivity to cyclooxygenase‐1 enzyme inhibitors [1, 2, 3]. The disease affects approximately 0.6%–2.5% of the general population, about 15% of patients with asthma, up to 16% of those with CRSwNP, and as many as 30% of patients with severe CRSwNP [3]. Asthma in N‐ERD has traditionally been associated with marked eosinophilic, type 2 (T2) inflammation [4]. However, more recent evidence indicates that N‐ERD is a heterogeneous disorder, albeit still predominantly characterized by T2‐driven airway inflammation [5, 6, 7]. The analysis of induced sputum cell profiles reveals the full spectrum of inflammatory asthma phenotypes: eosinophilic, neutrophilic, mixed granulocytic, and paucigranulocytic [5, 6, 7, 8]. The noneosinophilic asthma phenotype, which includes both paucigranulocytic and neutrophilic subtypes, is typically associated with non‐T2 asthma characterized by T helper 1 and 17 cytokine profiles, such as interferon γ and interleukins IL‐17 A/F, respectively [8].

The paucigranulocytic asthma phenotype is defined by the absence of elevated sputum eosinophils and neutrophils. Commonly accepted thresholds for this phenotype are < 2% or < 3% eosinophils and < 61% or < 64% neutrophils in sputum [8, 9, 10, 11]. Recent studies in the N‐ERD population have shown that up to 50% of patients exhibit a noneosinophilic inflammatory phenotype, with the paucigranulocytic pattern being the most prevalent, accounting for 75.8% of noneosinophilic cases [5, 6, 12].

The mechanisms underlying paucigranulocytic inflammation remain unclear but may involve asthma in remission, corticosteroid‐mediated suppression of airway eosinophilia, corticosteroid‐resistant airway inflammation, or airway remodeling [10, 13]. Macrophages have been reported as the predominant cell type in sputum from patients with the paucigranulocytic asthma phenotype [12, 14]. Recently, our team identified 4 distinct clusters among N‐ERD patients with noneosinophilic asthma inflammatory phenotype [12]. These clusters differed in inflammatory phenotypes, asthma severity, and prostaglandin D_2_ (PGD_2_) level, highlighting the heterogeneity of the noneosinophilic lower airway inflammation in N‐ERD [12].

The primary aim of this study was to further characterize the paucigranulocytic airway inflammatory phenotype in patients with N‐ERD. A secondary aim was to identify biochemical and imaging cut‐off values that differentiate T2 from non‐T2 inflammation within the paucigranulocytic phenotype, to guide the selection of appropriate biologic therapies.

## 2 Materials and Methods

### 2.1 Study Group

**FIGURE 1:**
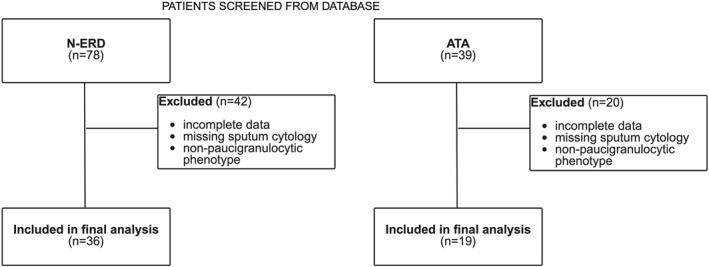
Patient selection flow diagram. Patients with N‐ERD and ATA were retrospectively screened from a database. Patients were excluded due to incomplete data, missing sputum cytology, or a non‐paucigranulocytic sputum inflammatory phenotype. Final cohorts included 36 N‐ERD patients and 19 ATA controls. ATA, aspirin‐tolerant asthma; N‐ERD, nonsteroidal anti‐inflammatory drug–exacerbated respiratory disease.

Participants were retrospectively identified from a database of patients with N‐ERD and aspirin‐tolerant asthma (ATA) diagnosed and treated at the Department of Internal Medicine, Jagiellonian University Medical College, Krakow, Poland. Data from 78 N‐ERD patients and 39 ATA controls, collected in our previous studies [5, 6, 12, 15, 16], were screened for eligibility. Asthma was defined according to GINA criteria as variable respiratory symptoms with documented bronchodilator reversibility after salbutamol [17]. N‐ERD was defined as a chronic inflammatory respiratory disorder in patients with asthma and/or CRSwNP, with respiratory symptoms exacerbated by NSAIDs; in this study, N‐ERD was confirmed by a positive oral aspirin provocation test [18, 19]. ATA was defined as asthma with a negative oral aspirin provocation test [19]. CRS and CRSwNP were defined as sinonasal symptoms lasting at least 12 weeks, with nasal polyps confirmed by an otolaryngologist in CRSwNP [20]. The patient selection process is presented in Figure 1. Patients were included if they had N‐ERD or ATA, as defined above, and a paucigranulocytic asthma phenotype determined by induced sputum cytology. Exclusion criteria were incomplete clinical or biomarker data; systemic corticosteroid or antihistamine use within 6 months before sputum sampling; prior biologic therapy; and respiratory tract infection or asthma exacerbation within 6 weeks before assessment. Of the screened population, 36 patients with N‐ERD (46%) and 19 ATA controls (49%) were included in the final analysis. All participants received ICS/LABA therapy for at least 6 weeks before sputum induction, and all had FEV1 ≥ 70% on the day of the procedure. Asthma severity was assessed according to GINA guidelines [17]. CRS was diagnosed by an otolaryngologist within 6 months before assessment; all patients with CRS received intranasal corticosteroids. Nasal symptoms were evaluated using SNOT‐22 [21], and sinus computed tomography scans were scored using the Lund–Mackay system [22]. Baseline characteristics are presented in Table 1. The study was conducted in accordance with the Declaration of Helsinki and approved by the Jagiellonian University Ethics Committee. Written informed consent was obtained from all participants.

**TABLE 1:** Baseline characteristics of patients before cluster analysis.

Variable		N‐ERD (*n* = 36)	ATA (*n* = 19)	*p*‐value
Age, years		46.3 (12.2) [24–73]	49.6 (9.9) [29–63]	0.293
Sex	Female	30 (83.3)	14 (73.7)	0.395*
	Male	6 (16.7)	5 (26.3)	
BMI, kg/m^2^		26.5 (4.5) [19.7–41.4]	26.7 (4.0) [20.2–35.9]	0.874
Asthma severity	Mild	4 (11.1)	6 (31.6)	0.083*
	Moderate	13 (36.1)	8 (42.1)	
	Severe	19 (52.8)	5 (26.3)	
ACT score		22.0 (19.0–23.3) [13–25]	24.0 (22.0–25.0) [17–25]	0.015
Disease duration, years		10.5 (6.8–15.8) [0–38]	9.0 (5.0–19.0) [1–34]	0.425
FEV_1_, % predicted		96.9 (13.1) [72–123]	105.2 (15.8) [83–144]	0.061
FEV_1_, mL		2825.3 (639.0) [1360–4320]	2811.2 (970.3) [2200–4170]	0.949
Positive skin prick test		15 (41.7)	10 (52.6)	0.437*
ICS dose, μg/day fluticasone eq.		800.0 (400.0–1000.0) [100–2000]	400.0 (112.5–665.0) [100–1100]	0.013
Blood eosinophils, cells/mm^3^		320.0 (257.5–438.3) [10–960]	300.0 (200.0–390.0) [33–740]	0.410
Total immunoglobulin E, IU/mL		112.5 (36.3–229.0) [16.2–1450.0]	60.5 (21.3–135.3) [16.5–304.0]	0.147
Sputum cells, %	Macrophages	52.9 (33.3–67.2) [8.0–88.0]	41.5 (31.1–61.9) [1.0–70.0]	0.296
	Lymphocytes	1.0 (0.5–2.2) [0.0–6.3]	1.1 (0.5–3.7) [0.0–9.3]	0.439
	Neutrophils	22.2 (10.8–46.3) [0.0–62.0]	40.3 (19.9–51.6) [2.8–61.7]	0.098
	Eosinophils	0.5 (0.0–0.9) [0.0–2.6]	0.9 (0.5–1.3) [0.0–2.7]	0.077
Chronic rhinosinusitis		36 (100)	16 (84.2)	0.068*
SNOT‐22 score		45.1 (17.0) [8–78]	24.8 (13.3) [6–53]	< 0.001
Lund‐Mackay score		15.0 (10.5–19.0) [0–24]	11.0 (5.0–13.0) [1–16]	0.004
ISS eicosanoids, pg/mL	LTC_4_	3.8 (1.2–36.7) [0.8–82.8]	2.4 (1.2–8.1) [0.9–120.6]	0.336
	LTD_4_	21.1 (13.1–49.2) [5.2–221.1]	28.2 (16.2–54.5) [2.9–180.5]	0.825
	LTE_4_	28.4 (17.4–106.1) [1.2–1193.5]	25.3 (10.8–68.1) [2.9–207.3]	0.269
	PGD_2_	50.3 (22.5–94.3) [7.5–323.9]	53.4 (31.0–80.7) [8.2–230.4]	0.070
	PGE_2_	104.3 (59.9–397.0) [15.2–2112.2]	58.3 (31.6–213.1) [15.5–334.0]	0.937
Urinary LTE_4_, pg/mg creatinine		428.7 (209.6–697.6) [18.0–3153.2]	327.0 (162.7–739.5) [88.1–1525.0]	0.730

*Note:* Data are presented as mean (SD) for normally distributed continuous variables, median (Q1–Q3) for non‐normally distributed continuous variables, and *n* (%) for categorical variables. Values in square brackets indicate min–max for continuous variables. Student’s *t*‐test was used for normally distributed continuous variables, the Mann–Whitney *U* test for non‐normally distributed variables, and Fisher’s exact test for categorical variables, as denoted by an asterisk. A *p*‐value < 0.05 was considered statistically significant. Red‐colored values indicate statistically significant differences at *p* < 0.05.

Abbreviations: ACT, Asthma Control Test; ATA, aspirin‐tolerant asthma; BMI, body mass index; FEV_1_, forced expiratory volume in one second; ICS, inhaled corticosteroid; ISS, induced sputum supernatant; LTC_4_, leukotriene C_4_; LTD_4_, leukotriene D_4_; LTE_4_, leukotriene E_4_; N‐ERD, nonsteroidal anti‐inflammatory drug–exacerbated respiratory disease; PGD_2_, prostaglandin D_2_; PGE_2_, prostaglandin E_2_; SNOT‐22, 22‐item Sino‐Nasal Outcome Test.

### 2.2 Study Design

Two independent cluster analyses were performed separately for the N‐ERD and ATA groups. A total of 23 variables (3 qualitative and 20 quantitative ones) were assessed, including the following: (1) demographic variables: age and sex; (2) clinical variables: body mass index (BMI), asthma severity, number of exacerbations in the past year, skin prick test results, forced expiratory volume in one second (FEV_1_), and Asthma Control Test (ACT) score; (3) treatment‐related variables: ICS dose; (4) blood parameters: eosinophil count and total serum immunoglobulin E (IgE); (5) sputum cytology: percentages of eosinophils, neutrophils, macrophages, and lymphocytes; (6) sinonasal disease measures: 22‐item Sino‐Nasal Outcome Test (SNOT‐22) score and Lund‐Mackay (LM) score based on paranasal sinus computed tomography (CT); (7) eicosanoid levels in induced sputum supernatant (ISS): leukotrienes LTC_4_, LTD_4_, and LTE_4_, as well as prostaglandins PGD_2_ and PGE_2_; and (8) urinary biomarker: LTE_4_ concentration.

### 2.3 Induced Sputum Collection and Inflammatory Phenotypes Based on Sputum Cell Counts

Induced sputum was collected according to the European Respiratory Society recommendations [23]. Differential cell counts were expressed as percentages based on a count of 800 inflammatory cells. The paucigranulocytic inflammatory phenotype was defined as a sputum eosinophil percentage below 3% and neutrophil percentage below 64% of inflammatory cells [8, 10, 11, 12].

### 2.4 Sputum and Urine Eicosanoids

Concentrations of LTC_4_, LTD_4_, LTE_4_, PGD_2_ and PGE_2_ in ISS were measured by high‐performance liquid chromatography–tandem mass spectrometry (HPLC‐MS/MS) (AB Sciex, Triple Quat 5500+), as previously described [5, 6, 12]. Results were expressed in picograms per milliliter. Urinary LTE_4_ concentrations were measured using HPLC‐MS/MS (AB Sciex, QTrap 4000), as previously described [5, 6, 12]. Results were expressed in picograms per milligram of creatinine.

### 2.5 T2 Inflammatory Profile Assessment

Participants with N‐ERD were classified as T2‐high or T2‐low based on three biomarkers: blood eosinophil count, LM score, and ISS LTE_4_. Thresholds for classification were determined during analysis. These markers were chosen as surrogate indicators of T2 inflammation, as IL‐4, IL‐5, IL‐13 and fractional exhaled nitric oxide, were not available in this retrospective study.

### 2.6 Statistical Analysis

Descriptive statistics for demographic, clinical, and laboratory variables were reported as mean (SD) or median (Q1–Q3) for continuous variables, and as frequencies and percentages for categorical variables. Student's *t*‐test was used for normally distributed continuous variables, whereas the Mann–Whitney *U* test for non‐normally distributed variables. Categorical variables were compared using Fisher's exact test. Multiple comparisons were corrected using the Benjamini–Hochberg false discovery rate method. Cut‐off values were determined using Youden’s index. A *p*‐value of < 0.05 was considered statistically significant.

Hierarchical cluster analysis was performed separately for the N‐ERD and ATA groups. Because the dataset included both quantitative and qualitative variables, factor analysis of mixed data (FAMD), with appropriate scaling of quantitative and qualitative variables, was first used for dimensionality reduction, retaining dimensions explaining at least 80% of the cumulative variance [24, 25]. Clustering was then performed on the retained dimensions using Euclidean distance and Ward's linkage method. The final number of clusters was selected based on NbClust recommendation and silhouette analysis [26]. Clustering tendency was assessed using the Hopkins statistic and the Visual Assessment of Cluster Tendency algorithm [27, 28]. Cluster validity and stability were evaluated using silhouette analysis, the cophenetic correlation coefficient, and pvClust [29]. Statistical analyses were conducted using Statistica version 13.3 (TIBCO Software Inc., Santa Clara, USA) and R packages FactoMineR, factoextra, NbClust, fpc, and pvClust [24, 25, 26, 29, 30, 31].

## 3 Results

### 3.1 Baseline Characteristics of the Study Groups

There were no significant differences between the N‐ERD and ATA groups in age, sex, BMI, asthma severity, or sputum cytology. However, compared with ATA controls, patients with N‐ERD had significantly lower ACT scores (22.0 vs. 24.0; *p* = 0.015), were treated with higher doses of ICS (800 vs. 400 μg fluticasone propionate or equivalent per day; *p* = 0.013), had higher SNOT‐22 scores (45.1 vs. 24.8; *p* < 0.001), and showed more severe sinonasal disease on CT (LM score: 15.0 vs. 11.0; *p* = 0.004). Detailed data are presented in Table 1.

### 3.2 Clustering Outcomes

**FIGURE 2:**
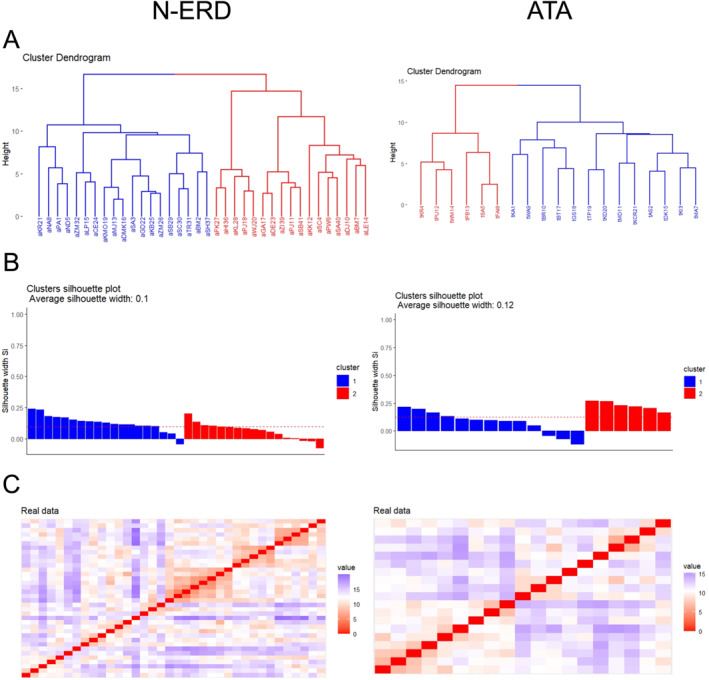
Cluster analysis results for patients with N‐ERD (left panels) and ATA (right panels). (A) Dendrograms of hierarchical clustering; (B) silhouette plots indicating average silhouette width of 0.1 for N‐ERD and 0.12 for ATA; (C) Euclidean distance matrices. ATA, aspirin‐tolerant asthma; N‐ERD, nonsteroidal anti‐inflammatory drug–exacerbated respiratory disease.

In each group, two clusters were identified: cluster 1_N‐ERD_ and cluster 2_N‐ERD_ among patients with N‐ERD, and cluster 1_ATA_ and cluster 2_ATA_ among patients with ATA. Cluster validity was assessed using silhouette analysis, with average silhouette widths of 0.10 for the N‐ERD group and 0.12 for the ATA group. For N‐ERD, the Hopkins statistic for real data was 0.647 and the cophenetic correlation coefficient (*R*) was 0.429. For ATA, the Hopkins statistic was 0.425 and *R* was 0.487. These values indicate weak cluster separation. The graphical representation of clustering outcomes is shown in Figure 2.

### 3.3 Characteristics of N‐ERD Clusters

**FIGURE 3:**
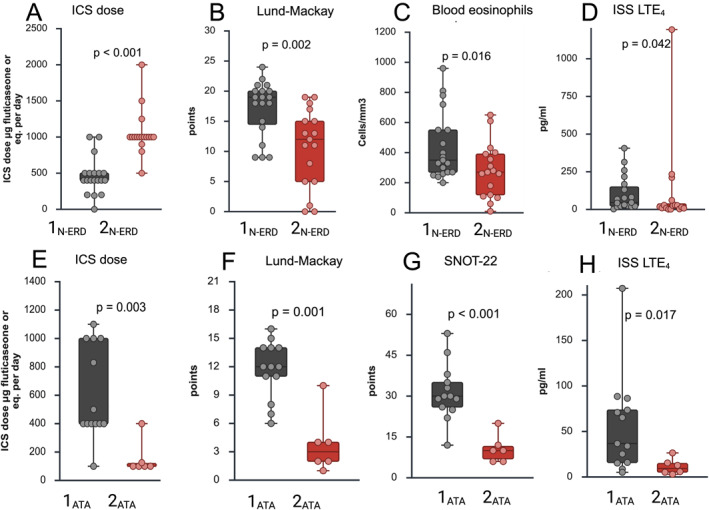
Differences between N‐ERD and ATA clusters. N‐ERD clusters (upper panels): (A) ICS dose (μg/day fluticasone or equivalent); (B) Lund‐Mackay score; (C) blood eosinophil count (cells/mm^3^); (D) LTE_4_ concentration in ISS (pg/mL). ATA clusters (lower panels): (E) ICS dose (μg/day fluticasone or equivalent); (F) Lund‐Mackay score; (G) SNOT‐22 score; (H) LTE_4_ concentration in ISS (pg/mL). ATA, aspirin‐tolerant asthma; ICS, inhaled corticosteroids; ISS, induced sputum supernatant; LTE4, leukotriene E4; N‐ERD, nonsteroidal anti‐inflammatory drug–exacerbated respiratory disease; SNOT‐22, 22‐item Sino‐Nasal Outcome Test.

Cluster 1_N‐ERD_ included 19 patients, while cluster 2_N‐ERD_ included 17 patients. Compared with cluster 2_N‐ERD_, patients in cluster 1_N‐ERD_ had less severe asthma (15.8% vs. 94.1% classified as severe; *p* < 0.001), required lower doses of ICSs (400 vs. 1000 μg of fluticasone propionate or equivalent per day; *p* < 0.001), and had higher blood eosinophil count (350.0 vs. 270 cells/mm^3^; *p* = 0.016). They also exhibited higher LM scores on sinus CT (19.0 vs. 12.0; *p* = 0.002) and higher LTE_4_ concentrations in ISS (45.7 vs. 21.6 pg/mL; *p* = 0.042). The time from the last sinus surgery to CT evaluation was significantly longer in cluster 1_N‐ERD_ compared to cluster 2_N‐ERD_ (*p* = 0.010). Patients in cluster 2_N‐ERD_ tended to have undergone more sinus surgeries, although the difference did not reach statistical significance (*p* = 0.067). No significant differences were observed between the clusters in terms of age, sex, BMI, asthma control, FEV_1_, total serum IgE, or skin prick test results. Additionally, there were no differences in sputum cytology, other eicosanoid concentrations in ISS, or urinary LTE_4_ levels. Further details are presented in Table 2, Figures 2 and 3.

**TABLE 2:** Differences between clusters in the N‐ERD group.

Variable		Cluster 1_N‐ERD_ (*n* = 19)	Cluster 2_N‐ERD_ (*n* = 17)	*p*‐value
Age, years		43.0 (36.0–56.0)	50.0 (45.0–55.0)	0.762
Sex	Female	17 (89.5)	13 (76.5)	0.391*
	Male	2 (10.5)	4 (23.5)	
BMI, kg/m^2^		24.6 (22.5–28.7)	25.9 (24.6–29.0)	0.502
Asthma severity	Mild	4 (21.0)	0	< 0.001*
	Moderate	12 (63.2)	1 (5.9)	
	Severe	3 (15.8)	17 (94.1)	
ACT score		23.0 (19.0–25.0)	21.0 (19.0–22.0)	0.225
FEV_1_, % predicted		96.9 (90.0–105.0)	102.0 (85.3–109.0)	0.491
Positive skin prick tests		7 (36.8)	8 (47.1)	0.736*
ICS dose, μg/day fluticasone eq.		400.0 (400.0–500.0)	1000.0 (1000.0–1000.0)	< 0.001
Blood eosinophils/mm^3^		350.0 (270.0–550.0)	270.0 (120.0–390.0)	0.016
Total immunoglobulin E, IU/mL		107.0 (31.1–150.0)	210.0 (38.0–339.0)	0.227
Sputum cells, %	Eosinophils	0.8 (0.0–1.9)	0.4 (0.0–0.8)	0.146
	Neutrophils	25.8 (10.3–49.8)	16.8 (11.0–44.8)	0.511
	Macrophages	52.6 (27.5–66.5)	53.1 (38.0–71.4)	0.545
	Lymphocytes	0.8 (0.5–2.1)	1.0 (0.7–2.3)	0.382
Chronic rhinosinusitis		19 (100)	17 (100)	1.0*
SNOT‐22 score		48.0 (35.0–59.0)	45.0 (33.0–55.0)	0.273
Lund‐Mackay score		19.0 (14.0–20.0)	12.0 (5.0–15.0)	0.002
Number of sinus surgeries		1.0 (1.0–2.0)	2.0 (2.0–3.0)	0.067
Time from last sinus surgery to CT (months)		17.0 (15.0–20.0)	12.0 (10.5–16.5)	0.010
ISS eicosanoids, pg/mL	LTC_4_	3.2 (1.2–24.1)	27.7 (1.7–46.6)	0.208
	LTD_4_	26.6 (16.5–57.0)	17.1 (7.6–45.2)	0.234
	LTE_4_	45.7 (22.4–149.3)	21.6 (8.8–36.6)	0.042
	PGD_2_	59.9 (35.8–95.6)	34.7 (15.3–81.1)	0.055
	PGE_2_	121.2 (90.1–466.4)	94.0 (37.7–331.0)	0.328
Urinary LTE_4_, pg/mg creatinine		450.3 (252.5–622.7)	407.2 (170.2–943.3)	0.888

*Note:* Data are presented as median (Q1–Q3) for continuous variables and *n* (%) for categorical variables. The Mann–Whitney *U* test was used to compare continuous variables between cluster 1_N‐ERD_ and cluster 2_N‐ERD_. Fisher's exact test was used for categorical variables, as denoted by an asterisk. A *p*‐value < 0.05 was considered statistically significant. Red‐colored values indicate statistically significant differences at *p* < 0.05.

Abbreviations: ACT, Asthma Control Test; BMI, body mass index; FEV_1_, forced expiratory volume in one second; ICS, inhaled corticosteroid; ISS, induced sputum supernatant; LTC_4_, leukotriene C_4_; LTD_4_, leukotriene D_4_; LTE_4_, leukotriene E_4_; N‐ERD, nonsteroidal anti‐inflammatory drug–exacerbated respiratory disease; PGD_2_, prostaglandin D_2_; PGE_2_, prostaglandin E_2_; SNOT‐22, Sino‐Nasal Outcome Test‐22.

### 3.4 Characteristics of ATA Clusters

Cluster 1_ATA_ included 13 patients, while cluster 2_ATA_ included 6 patients. Compared with cluster 2_ATA_, patients in cluster 1_ATA_ had more severe asthma (38.5% of severe asthma vs. 0%; *p* = 0.004) and required higher doses of ICSs (400 vs. 100 μg of fluticasone or equivalent per day; *p* = 0.003). CRSwNP was present in all patients in cluster 1_ATA_ but only in half of those in cluster 2_ATA_ (100% vs. 50%; *p* = 0.021). Patients in cluster 1_ATA_ also reported more severe CRSwNP symptoms, as indicated by higher SNOT‐22 scores (30.0 vs. 10.0; *p* < 0.001), and showed more advanced sinonasal disease on CT, with higher LM scores (12.0 vs. 3.0; *p* = 0.001). Inflammatory marker analysis revealed higher concentrations of LTD_4_ (38.1 vs. 17.7 pg/mL; *p* = 0.036) and LTE_4_ (36.7 vs. 9.4 pg/mL; *p* = 0.017) in ISS in cluster 1_ATA_. No significant differences were observed in age, sex, BMI, asthma control, FEV_1_, total serum IgE, and skin prick test results. Additionally, there were no differences in sputum cytology, other ISS eicosanoids, or urinary LTE_4_ concentrations. Further details are presented in Table 3, Figures 2 and 3.

**TABLE 3:** Differences between clusters in the ATA group.

Variable		Cluster 1_ATA_ (*n* = 13)	Cluster 2_ATA_ (*n* = 6)	*p*‐value
Age, years		49.0 (42.0–56.0)	54.0 (41.0–60.0)	0.765
Sex	Female	8 (61.5)	6 (100)	0.128*
	Male	5 (38.5)	0 (0)	
BMI, kg/m^2^		26.7 (24.1–27.4)	26.4 (24.1–29.2)	0.898
Asthma severity	Mild	1 (7.7)	5 (83.3)	0.004*
	Moderate	7 (53.8)	1 (16.7)	
	Severe	5 (38.5)	0 (0)	
ACT score		24.0 (22.0–25.0)	24.5 (20.0–25.0)	0.966
FEV_1_, % predicted		103.6 (92.0–109.7)	109.5 (103.4–112.9)	0.639
Positive skin prick tests		6 (46.2)	4 (66.7)	0.628*
ICS dose, μg/day fluticasone eq.		400.0 (400.0–1000.0)	100.0 (100.0–125.0)	0.003
Blood eosinophils/mm^3^		310.0 (200.0–410.0)	296.0 (243.0–280.0)	0.831
Total immunoglobulin E, IU/mL		51.1 (22.6–112.7)	129.6 (17.1–254.0)	0.467
Sputum cells, %	Eosinophils	1.0 (0.8–2.1)	0.5 (0.0–1.2)	0.127
	Neutrophils	40.4 (15.0–51.0)	41.0 (34.1–54.5)	0.416
	Macrophages	41.5 (25.0–62.5)	50.7 (37.3–61.2)	1.0
	Lymphocytes	0.8 (0.5–1.3)	3.0 (1.2–4.1)	0.179
Chronic rhinosinusitis		13 (100)	3 (50)	0.021*
SNOT‐22 score		30.0 (26.0–35.0)	10.0 (6.0–12.0)	< 0.001
Lund‐Mackay score		12.0 (11.0–14.0)	3.0 (2.0–4.0)	0.001
ISS eicosanoids, pg/mL	LTC_4_	1.2 (1.2–3.9)	2.9 (2.6–26.0)	0.058
	LTD_4_	38.1 (21.9–63.8)	17.7 (9.5–19.1)	0.036
	LTE_4_	36.7 (16.0–73.5)	9.4 (5.4–14.8)	0.017
	PGD_2_	62.4 (35.2–108.7)	40.2 (17.7–53.0)	0.106
	PGE_2_	150.3 (33.1–258.0)	38.7 (31.0–52.4)	0.072
Urinary LTE_4_, pg/mg creatinine		327.0 (165.4–721.0)	468.5 (163.0–818.3)	0.831

*Note:* Data are presented as median (Q1–Q3) for continuous variables and *n* (%) for categorical variables. The Mann–Whitney *U* test was used to compare continuous variables between cluster 1_ATA_ and cluster 2_ATA_. Fisher's exact test was used for categorical variables, as denoted by an asterisk. A *p*‐value < 0.05 was considered statistically significant. Red‐colored values indicate statistically significant differences at *p* < 0.05.

Abbreviations: ACT, Asthma Control Test; ATA, aspirin‐tolerant asthma; BMI, body mass index; FEV_1_, forced expiratory volume in one second; ICS, inhaled corticosteroid; ISS, induced sputum supernatant; LTC_4_, leukotriene C_4_; LTD_4_, leukotriene D_4_; LTE_4_, leukotriene E_4_; PGD_2_, prostaglandin D_2_; PGE_2_, prostaglandin E_2_; SNOT‐22, Sino‐Nasal Outcome Test‐22.

### 3.5 Exploratory Comparison of N‐ERD and ATA Cluster Patterns

An exploratory comparison of all four independently derived clusters is presented in Supporting Information S1: Supplementary Results and Table S1.

### 3.6 Correlations in N‐ERD and ATA Groups

In the overall N‐ERD group, a positive correlation was observed between blood eosinophil count and LM score (*r* = 0.47, *p* = 0.004), which was also present in cluster 2_N‐ERD_ (*r* = 0.578, *p* = 0.014), but not in cluster 1_N‐ERD_. Conversely, a positive correlation between blood eosinophil count and ICS dose was found in cluster 1_N‐ERD_ (*r* = 0.64, *p* = 0.003), but not in cluster 2_N‐ERD_ or the overall N‐ERD group. No significant correlations were found between blood eosinophil count and ICS dose in the overall ATA group or within ATA clusters.

### 3.7 Exploratory Cut‐Off Values for a T2‐High‐Like Profile in the N‐ERD Group

**FIGURE 4:**
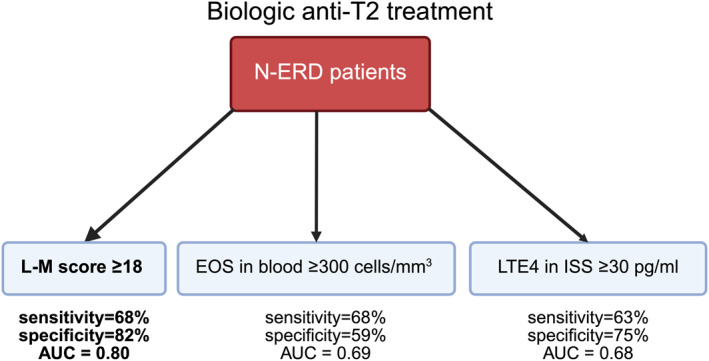
Identification of optimal cut‐off values for three biomarkers identifying a T2‐high‐like profile in N‐ERD patients: the Lund‐Mackay score, blood eosinophil count, and LTE_4_ levels in ISS. The highest AUC was observed for the LM score. AUC, area under the curve; EOS, eosinophils; ISS, induced sputum supernatant; LM score, Lund‐Mackay score based on computed tomography of paranasal sinuses; LTE4, leukotriene E4; N‐ERD, nonsteroidal anti‐inflammatory drug–exacerbated respiratory disease.

Using Youden’s index, optimal cut‐off values were determined for three biomarkers to identify a T2‐high‐like profile in patients with N‐ERD. The selected biomarkers and their respective cut‐off values included the LM score (≥ 18), blood eosinophil count (≥ 300 cells/mm^3^), or LTE_4_ levels in ISS (≥ 30 pg/mL). The corresponding sensitivity, specificity, and area under the curve (AUC) values for each biomarker are presented in Figure 4.

Notably, combining these biomarkers did not enhance diagnostic performance, as no combination yielded higher sensitivity or specificity than the individual parameters. Among the three, the LM score demonstrated the highest AUC, indicating that the extent of sinonasal disease was the strongest single predictor of a T2‐high inflammatory profile in this cohort.

## 4 Discussion

To the best of our knowledge, this is the first study to identify subtypes of paucigranulocytic asthma within N‐ERD using cluster analysis. Previous clustering studies have been limited to the general asthma population [11]. In this study, hierarchical cluster analysis identified two clusters in both the N‐ERD and ATA groups: 1_N‐ERD_, 2_N‐ERD_ and 1_ATA_, 2_ATA_, respectively. These clusters categorized patients with or without aspirin hypersensitivity based on similarities across 23 variables, including demographic, clinical, treatment, radiologic, and biochemical parameters. The ATA group was included as a comparator to provide clinical and biochemical context for the N‐ERD findings and to assess whether similar patterns could be observed in aspirin‐tolerant patients with a comparable paucigranulocytic phenotype.

At baseline, patients with N‐ERD and ATA did not differ in age, sex, BMI, or asthma severity. Unexpectedly, there were also no differences in sputum cytology, including macrophage percentages, or in urinary LTE_4_ levels. However, prior studies have demonstrated that patients with N‐ERD, across various bronchial inflammatory phenotypes, typically exhibit significantly higher urinary LTE_4_ levels compared with asthmatics without aspirin hypersensitivity [32, 33].

We hypothesized that the lack of differences in urinary LTE_4_ levels observed in our study groups may be related to the predominance of the paucigranulocytic sputum phenotype, which is characterized by low numbers of airway inflammatory cells responsible for cysteinyl leukotriene production and/or altered polarization of airway immune cells toward a T2‐associated inflammatory profile [34]. Interestingly, no differences in systemic LTE_4_ production were observed, despite poorer asthma control, higher ICS doses, and more severe sinonasal disease in patients with N‐ERD, as reflected by LM and SNOT‐22 scores, which is consistent with previous reports [35, 36].

Although statistically significant differences were observed between clusters, the cluster validity metrics suggest that the identified clusters should be interpreted with caution and may reflect a continuum of disease severity rather than clearly distinct biological subtypes. The cluster analysis in the N‐ERD group yielded unexpected results. Patients in cluster 1_N‐ERD_ had less severe asthma and required lower doses of ICSs, yet paradoxically exhibited higher blood eosinophil counts, more severe sinonasal disease as indicated by elevated LM scores, and increased LTE_4_ concentrations in ISS. However, there were no differences between clusters 1_N‐ERD_ and 2_N‐ERD_ in sputum cytology or systemic urinary LTE_4_ excretion. These findings suggest a dissociation between asthma severity, sinonasal disease severity, and both local and systemic cysteinyl leukotriene production. The lower LM scores observed in cluster 2_N‐ERD_ may be attributable to the shorter interval between the most recent sinus surgery and imaging, compared with cluster 1_N‐ERD_. Additionally, the higher blood eosinophil count in cluster 1_N‐ERD_ may reflect more severe underlying sinus inflammation [37]. Indeed, most patients with N‐ERD exhibit eosinophilic infiltration in nasal polyp tissue [38]. However, in cluster 1_N‐ERD_, no significant correlation was observed between the LM score and blood eosinophil count. In contrast, a significant positive correlation between these variables was found in the overall N‐ERD group and in cluster 2_N‐ERD_, suggesting that the relationship between systemic eosinophilia and sinonasal disease severity may vary across N‐ERD subgroups.

In our cohort, sputum eosinophil percentages did not differ between the N‐ERD clusters, suggesting that elevated ISS LTE_4_ in cluster 1_N‐ERD_ may reflect increased eosinophil activation rather than higher eosinophil numbers, consistent with enhanced mediator release by eosinophils in N‐ERD [39]. This interpretation is supported by the weak correlation between blood and sputum eosinophils [40, 41] and by evidence that inflammatory CD62L‐low eosinophils are increased in severe asthma, correlate with disease severity, and may be linked to differential responses to mepolizumab [42, 43, 44]. Although CD62L expression was not assessed in our study, functional eosinophil activity, together with other sputum cell populations such as basophils, alternatively activated macrophages, or airway epithelial cells, may contribute to elevated ISS LTE_4_ in cluster 1_N‐ERD_ [34].

The paucigranulocytic asthma phenotype may not be truly hypocellular, as macrophages can predominate when eosinophils and neutrophils are not elevated [12, 14]. In N‐ERD, alveolar‐like monocyte‐derived macrophages may undergo sustained epigenetic and metabolic reprogramming, with increased production of 5‐lipoxygenase‐derived metabolites and upregulation of T2‐related chemokine and cytokine genes [34]. Such macrophage activation may contribute to therapy‐resistant airway inflammation [45]. M2‐polarized macrophages promote Th2 lymphocyte and eosinophil recruitment [46], and SERPINB10 has been implicated in M2 polarization [47]. We therefore hypothesize that elevated blood eosinophils in cluster 1_N‐ERD_ may promote macrophage activation with T2 features, thereby increasing local LTE_4_ production, although macrophage phenotypes were not assessed in this study.

The division of ATA patients into two clusters appears to show clinically relevant patterns. Patients in cluster 1_ATA_ had more severe asthma, required higher doses of ICSs, and exhibited more severe sinonasal disease, as indicated by elevated SNOT‐22 and LM scores on CT. They also showed higher sputum concentrations of LTD_4_ and LTE_4_ in ISS compared with cluster 2_ATA_. However, blood eosinophil counts and sputum inflammatory cell profiles did not differ significantly between clusters. In contrast, cluster 2_ATA_ was characterized by milder asthma and lower corticosteroid requirements. Urinary LTE_4_ levels were similar in both clusters. These findings suggest that local sputum concentrations of 5‐LO–derived metabolites may serve as more informative biomarkers of the paucigranulocytic phenotype than systemic urinary LTE_4_ levels. Additionally, elevated sputum PGD_2_ in cluster 1_ATA_ may reflect ongoing mast cell activation, consistent with previous reports [12, 48].

The exploratory post hoc comparison presented in Supporting Information S1 showed that the main differences across N‐ERD and ATA cluster patterns involved asthma severity, treatment intensity, sinonasal disease severity, and local eicosanoid production. In contrast, sputum cytology and urinary LTE_4_ did not differ across the four clusters.

An additional objective of this study was to identify biochemical and imaging cut‐off values to distinguish T2 from non‐T2 inflammation and support the selection of candidates for targeted biologic therapy. Patients in cluster 1_N‐ERD_ exhibited elevated blood eosinophil counts and higher LTE_4_ levels in ISS compared to those in cluster 2_N‐ERD_, suggesting a T2‐high‐like inflammatory profile. These findings may help identify patients who could be considered for further evaluation for biologic therapy primarily due to severe CRSwNP [1, 49], potentially meeting treatment criteria for agents such as dupilumab [50] based on CRSwNP rather than asthma severity. Cut‐off values identified using Youden's index included a Lund‐Mackay score ≥ 18, blood eosinophil count ≥ 300 cells/mm^3^, and LTE_4_ concentration in ISS ≥ 30 pg/mL. Among these, the LM score demonstrated the highest discriminatory performance and may serve as the most informative single marker supporting further evaluation for T2‐targeted biologic therapy.

Clinically, these findings suggest that a paucigranulocytic sputum phenotype should not be interpreted as excluding residual T2‐high‐like inflammation in N‐ERD. In patients with low sputum eosinophil counts, severe sinonasal disease, elevated blood eosinophils, and increased local LTE_4_ production may indicate persistent T2‐spectrum activity, particularly in the upper airway compartment. This may help identify patients who require closer otolaryngologic and allergy assessment, even when asthma severity alone would not suggest eligibility for biologic therapy [1, 49, 50, 51]. Thus, combining lower airway cytology with upper airway imaging and systemic biomarkers may improve patient stratification in paucigranulocytic N‐ERD. However, the proposed cut‐off values should be considered exploratory and require prospective validation before they can be used in routine clinical decision‐making.

This study has several limitations. First, the retrospective design and lack of T1, T2, and T3 cytokine profiling in blood and sputum supernatant limited detailed characterization of inflammatory patterns in the paucigranulocytic asthma phenotype. Second, plasma LTE_4_ was not measured because of its rapid metabolic degradation; therefore, urinary LTE_4_ was used as a stable metabolite. Third, the presence of nasal polyps in ATA patients with CRS could not be retrospectively confirmed, whereas all N‐ERD patients had CRSwNP. Fourth, prior sinus surgeries may have influenced LM scores and therefore the assessment of sinus disease severity. Fifth, the T2‐high‐like inflammatory profile was defined using single time‐point biomarkers, including blood eosinophil count; therefore, its temporal stability should be confirmed in future prospective studies with repeated eosinophil and mediator assessments. Sixth, although ATA patients with a comparable paucigranulocytic asthma phenotype were included as a clinical comparator group, the study did not include healthy controls; therefore, comparisons with non‐asthmatic individuals were not possible and should be addressed in future studies. Finally, future studies should evaluate sputum macrophage polarization toward a T2 phenotype and functional eosinophil activity, in addition to cell counts, to better understand cluster‐specific mediator profiles [34, 41, 42, 43, 44].

Cluster analysis of paucigranulocytic asthma in N‐ERD revealed two phenotypic subgroups. Cluster 1_N‐ERD_ was characterized by milder asthma, higher blood eosinophil counts, more severe sinonasal disease, and elevated LTE_4_ levels in ISS, despite comparable sputum cytology. These findings suggest that a paucigranulocytic sputum phenotype may coexist with features of T2‐spectrum inflammation, particularly in the upper airways. Assessment of sinonasal disease severity, blood eosinophils, and local eicosanoid production may therefore support patient stratification and help identify individuals requiring further evaluation for T2‐targeted therapy. However, these observations remain exploratory and require prospective validation.

## Author Contributions

**Radosław Kacorzyk:** methodology, data curation, writing – review and editing, writing – original draft, visualization, resources, conceptualization, investigation, formal analysis. **Piotr Szatkowski:** data curation, writing – review and editing, methodology, resources, formal analysis. **Agnieszka Gawlewicz‐Mroczka:** writing – review and editing, supervision. **Adam Ćmiel:** methodology, validation, formal analysis, software, visualization. **Anna Gielicz:** data curation, resources. **Adam Stępień:** data curation, resources. **Patryk Hartwich:** data curation, resources. **Marek Sanak:** supervision, writing – review and editing. **Lucyna Mastalerz:** conceptualization, investigation, funding acquisition, methodology, writing – review and editing, supervision, writing – original draft, project administration.

## Funding

This work was supported by National Science Centre, Poland (Grant 2020/39/B/NZ5/02296). The article processing charge was funded by the Visibility and Mobility Module 2026 within the Doctoral School of Medical and Health Sciences, Jagiellonian University Medical College, under the Visibility & Mobility Module within the Excellence Initiative at Jagiellonian University (ID.UJ).

## Conflicts of Interest

The authors declare no conflicts of interest.

## Supporting information


Supporting Information S1

## Data Availability

The data that support the findings of this study are available from the corresponding author upon reasonable request.
