# Supplementary material for: Paucigranulocytic Asthma in Aspirin‐Hypersensitive Patients: Pleiotropic Regulation of Type 2 Biomarkers
Source: Clin Transl Allergy. 2026 Jul 19;16(7):e70186. doi: 10.1002/clt2.70186 (PMC13381727; doi:10.1002/clt2.70186)
Supplement: Supplementary file 1 — Supporting Information S1 [file CLT2-16-e70186-s001.docx]

**Title:** Paucigranulocytic asthma in aspirin-hypersensitive patients: pleiotropic regulation of type 2 biomarkers

Supplementary Material

**Results**
**Exploratory comparison of N-ERD and ATA cluster patterns**

An exploratory comparison of all four clusters: 1_N-ERD_, 2_N-ERD_, 1_ATA_, and 2_ATA_ is presented in Supplementary Table S1. Significant differences across the four cluster patterns were observed for asthma severity, ACT score, ICS dose, SNOT-22 score, Lund-Mackay score, ISS LTE_4_, and ISS PGE_2_. No significant differences across the four clusters were found for age, sex, BMI, FEV1, skin prick test positivity, blood eosinophil count, total IgE, sputum eosinophil, neutrophil, macrophage or lymphocyte percentages, ISS LTC_4_, ISS LTD_4_, ISS PGD_2_, or urinary LTE_4_.

Post hoc analyses showed that differences in ACT score were mainly related to lower asthma control in cluster 2_N-ERD_ compared with cluster 1_ATA_. ICS dose differed mainly between cluster 2_N-ERD_ and both ATA clusters, particularly cluster 2_ATA_. SNOT-22 score was higher in cluster 1_N-ERD_ than in cluster 1_ATA_ and cluster 2_ATA_, and higher in cluster 2_N-ERD_ than in cluster 2_ATA_. Lund-Mackay score was higher in cluster 1_N-ERD_ than in cluster 1_ATA_ and cluster 2_ATA_, and higher in cluster 2_N-ERD_ than in cluster 2_ATA_. Among eicosanoid mediators, ISS LTE_4_ was significantly higher in cluster 1_N-ERD_ than in cluster 2_ATA_, and ISS PGE_2_ was also higher in cluster 1_N-ERD_ than in cluster 2_ATA_.

Overall, this exploratory analysis suggests that differences across the four cluster patterns were driven mainly by asthma severity and treatment intensity, sinonasal disease severity, and local eicosanoid production. In contrast, sputum cytology and urinary LTE_4_ did not significantly differ across the four clusters. Because the N-ERD and ATA clusters were derived from separate hierarchical cluster analyses, these post hoc comparisons should be interpreted cautiously and considered supportive rather than confirmatory.

**Table S1.** Exploratory comparison of N-ERD and ATA cluster patterns.

| **Variable** | | | | **Cluster 1_N-ERD_ (n=19)** | **Cluster 2_N-ERD_ (n=17)** | **p-value*** | **Cluster 1_ATA_ (n=13)** | **Cluster 2_ATA_(n=6)** | **p-value*** | **p-value**^α^ | **p-value post hoc** |
| --- | --- | --- | --- | --- | --- | --- | --- | --- | --- | --- | --- |
| Age, years | | | | 43.0 (36.0-56.0) | 50.0 (45.0-55.0) | .762 | 49.0 (42.0-56.0) | 54.0 (41.0-60.0) | .765 | .599 | - |
| Sex | | | Female | 17 (89.5) | 13 (76.5) | .391 | 8 (61.5) | 6 (100) | .128 | .141 | - |
|  |  |  | Male | 2 (10.5) | 4 (23.5) |  | 5 (38.5) | 0 (0) |  |  |  |
| BMI, kg/m^2^ | | | | 24.6 (22.5-28.7) | 25.9 (24.6-29.0) | .502 | 26.7 (24.1-27.4) | 26.4 (24.1-29.2) | .898 | .735 | - |
| Asthma severity | Mild | | | 4 (21.0) | 0 | <.001 | 1 (7.7) | 5 (83.3) | 0.004 | <.001 | - |
|  | Moderate | | | 12 (63.2) | 1 (5.9) |  | 7 (53.8) | 1 (16.7) |  |  |  |
|  | Severe | | | 3 (15.8) | 17 (94.1) |  | 5 (38.5) | 0 (0) |  |  |  |
| ACT score | | | | 23.0 (19.0-25.0) | 21.0 (19.0-22.0) | .225 | 24.0 (22.0-25.0) | 24.5 (20.0-25.0) | .966 | .023 | 2_N-ERD_ vs 1_ATA_: p=0.034 |
| FEV_1_, % predicted | | | | 96.9 (90.0-105.0) | 102.0 (85.3-109.0) | .491 | 103.6 (92.0-109.7) | 109.5 (103.4-112.9) | .639 | .246 | - |
| Positive skin prick tests | | | | 7 (36.8) | 8 (47.1) | .736 | 6 (46.2) | 4 (66.7) | .628 | .642 | - |
| ICS dose, μg/day fluticasone eq. | | | | 400.0 (400.0-500.0) | 1000.0 (1000.0-1000.0) | <.001 | 400.0 (400.0-1000.0) | 100.0 (100.0-125.0) | .003 | <.001 | 2_N-ERD_ vs 1_ATA_: p=.038  2_N-ERD_ vs 2_ATA_: p<.001 |
| Blood eosinophils/mm^3^ | | | | 350.0 (270.0-550.0) | 270.0 (120.0-390.0) | .016 | 310.0 (200.0-410.0) | 296.0 (243.0-280.0) | .831 | .162 | - |
| Total immunoglobulin E, IU/mL | | | | 107.0 (31.1-150.0) | 210.0 (38.0-339.0) | .227 | 51.1 (22.6-112.7) | 129.6 (17.1-254.0) | .467 | .268 | - |
| Sputum cells, % | | Eosinophils | | 0.8 (0.0-1.9) | 0.4 (0.0-0.8) | .146 | 1.0 (0.8-2.1) | 0.5 (0.0-1.2) | .127 | .064 | - |
|  |  | Neutrophils | | 25.8 (10.3-49.8) | 16.8 (11.0-44.8) | .511 | 40.4 (15.0-51.0) | 41.0 (34.1-54.5) | .416 | .228 | - |
|  |  | Macrophages | | 52.6 (27.5-66.5) | 53.1 (38.0-71.4) | .545 | 41.5 (25.0-62.5) | 50.7 (37.3-61.2) | 1.0 | .712 | - |
|  |  | Lymphocytes | | 0.8 (0.5-2.1) | 1.0 (0.7-2.3) | .382 | 0.8 (0.5-1.3) | 3.0 (1.2-4.1) | .179 | .247 | - |
| SNOT-22 score | | | | 48.0 (35.0-59.0) | 45.0 (33.0-55.0) | .273 | 30.0 (26.0-35.0) | 10.0 (6.0-12.0) | <.001 | <.001 | 1_N-ERD_ vs 1_ATA_: p=.048  1_N-ERD_ vs 2_ATA_: p<.001  2_N-ERD_ vs 2_ATA_: p=.003 |
| Lund‐Mackay score | | | | 19.0 (14.0-20.0) | 12.0 (5.0-15.0) | .002 | 12.0 (11.0-14.0) | 3.0 (2.0-4.0) | .001 | .001 | 1_N-ERD_ vs 1_ATA_: p=.048  1_N-ERD_ vs 2_ATA_: p<.001  2_N-ERD_ vs 2_ATA_: p=.014 |
| ISS eicosanoids, pg/mL | | | LTC_4_ | 3.2 (1.2-24.1) | 27.7 (1.7-46.6) | .208 | 1.2 (1.2-3.9) | 2.9 (2.6-26.0) | .058 | .112 | - |
|  |  |  | LTD_4_ | 26.6 (16.5-57.0) | 17.1 (7.6-45.2) | .234 | 38.1 (21.9-63.8) | 17.7 (9.5-19.1) | .036 | .103 | - |
|  |  |  | LTE_4_ | 45.7 (22.4-149.3) | 21.6 (8.8-36.6) | .042 | 36.7 (16.0-73.5) | 9.4 (5.4-14.8) | .017 | .017 | 1_N-ERD_ vs 2_ATA_: p=.019 |
|  |  |  | PGD_2_ | 59.9 (35.8-95.6) | 34.7 (15.3-81.1) | .055 | 62.4 (35.2-108.7) | 40.2 (17.7-53.0) | .106 | .159 | - |
|  |  |  | PGE_2_ | 121.2 (90.1-466.4) | 94.0 (37.7-331.0) | .328 | 150.3 (33.1-258.0) | 38.7 (31.0-52.4) | .072 | .028 | 1_N-ERD_ vs 2_ATA_: p=.016 |
| Urinary LTE_4_, pg/mg creatinine | | | | 450.3 (252.5-622.7) | 407.2 (170.2-943.3) | .888 | 327.0 (165.4-721.0) | 468.5 (163.0-818.3) | .831 | .976 | - |

*Differences between cluster 1_N-ERD_ vs cluster 2_N-ERD_ or between cluster 1_ATA_ vs cluster 2_ATA_ were considered significant at p < .05.

^α^Differences between all clusters: cluster 1_N-ERD_ cluster 2_N-ERD_, cluster 1_ATA_, and cluster 2_ATA_ were considered significant at p < .05.

Data are presented as median (Q1–Q3) for continuous variables and n (%) for categorical variables.

Abbreviations: ACT, Asthma Control Test; ATA, aspirin-tolerant asthma; BMI, body mass index; FEV_1_, forced expiratory volume in one second; ICS, inhaled corticosteroids; ISS, induced sputum supernatant; LM, Lund-Mackay; LTC_4_, leukotriene C_4_; LTD_4_, leukotriene D_4_; LTE_4_, leukotriene E_4_; N-ERD, nonsteroidal anti-inflammatory drug–exacerbated respiratory disease; PGD_2_, prostaglandin D_2_; PGE_2_, prostaglandin E_2_; SNOT-22, 22-item Sino-Nasal Outcome Test.
